# RCAN1 Overexpression Exacerbates Calcium Overloading-Induced Neuronal Apoptosis

**DOI:** 10.1371/journal.pone.0095471

**Published:** 2014-04-21

**Authors:** Xiulian Sun, Yili Wu, Bruno Herculano, Weihong Song

**Affiliations:** 1 Qilu Hospital of Shandong University, Jinan, China; 2 Chongqing City Key Lab of Translational Medical Research in Cognitive Development and Learning and Memory Disorders, and Ministry of Education Key Laboratory of Child Development and Disorders, Children's Hospital of Chongqing Medical University, Chongqing, China; 3 Townsend Family Laboratories, Department of Psychiatry, Brain Research Center, Graduate Program in Neuroscience, The University of British Columbia, Vancouver, Canada; Cleveland Clnic Foundation, United States of America

## Abstract

Down Syndrome (DS) patients develop characteristic Alzheimer's Disease (AD) neuropathology after their middle age. Prominent neuronal loss has been observed in the cortical regions of AD brains. However, the underlying mechanism leading to this neuronal loss in both DS and AD remains to be elucidated. Calcium overloading and oxidative stress have been implicated in AD pathogenesis. Two major isoforms of regulator of calcineurin 1 (RCAN1), RCAN1.1 and RCAN1.4, are detected in human brains. In this report we defined the transcriptional regulation of RCAN1.1 and RCAN1.4 by two alternative promoters. Calcium overloading upregulated RCAN1.4 expression by activating RCAN1.4 promoter through calcineurin-NFAT signaling pathway, thus forming a negative feedback loop in isoform 4 regulation. Furthermore, RCAN1.4 overexpression exacerbated calcium overloading-induced neuronal apoptosis, which was mediated by caspase-3 apoptotic pathway. Our results suggest that downregulating RCAN1.4 expression in neurons could be beneficial to AD patients.

## Introduction

Down Syndrome (DS) is caused by an extra copy of chromosome 21 and affects approximately one in 800 to 1,000 babies [1], [2], [3]. Individuals with DS inevitably develop neuropathological changes characteristic of Alzheimer's disease (AD) in the brain following their middle age, including neuritic plaques and neuronal loss, thus making DS a valuable model system for understanding AD pathogenesis. Among the possible factors, dysregulation of the regulatory circuit of regulator of calcineurin 1 (RCAN1)-calcineurin- nuclear factor of activated T-cells (NFAT) plays an important role in DS pathogenesis [4].

Calcineurin, also known as protein phosphatase 2B (PP2B), is a calcium/calmodulin-dependent serine/threonine phosphatase. It consists of a catalytic subunit calcineurin A and a regulatory subunit calcineurin B, forming a heterodimer, which is involved in many physiological processes, such as apoptosis and cardiovascular development. Calcineurin activates NFAT through dephosphorylation, which further alters NFAT-dependent gene transcription. As the most calcium sensitive (K_d_  =  0.1–1 nM) and the unique calcium-activated protein phosphatase in the brain [5], calcineurin is associated with learning and memory. RCAN1 is an endogenous regulator of calcineurin, therefore, dysregulations of RCAN1 may interrupt learning and memory. In support of this hypothesis, both disruption and overexpression of *nebula,* an RCAN1 ortholog in *Drosophila*, has been shown to lead to severe learning and memory deficits [6]. Moreover, RCAN1overexpression was seen to reduce the number of neurons in hippocampus, resulting in learning and memory deficits in RCAN1transgenic mice [7]. Furthermore, calcineurin can dephosphorylate hyperphosphorylated tau protein, accumulation of which is another pathological hallmark of AD. Inhibition of calcineurin by RCAN1 overexpression in AD may thus contribute to hyperphosphorylation and accumulation of tau, and subsequent formation of neurofibrillary tangles. Consistent with this, recent studies showed that induction of RCAN1 expression increases tau phosphorylation in PC12 cells, and tau hyperphosphorylation and neurofibrillary tangle formation in transgenic mice [8], [9]. These data suggest that apart from the pro-apoptotic effect of RCAN1 in AD pathogenesis, it may also contribute to AD pathogenesis by facilitating tau phosphorylation.

Initially identified in the DS critical region, the *RCAN1* gene spans about 45 kb of genome and has seven exons and six introns [10]. Alternative splicing of the first four exons generates four isoforms of RCAN1 that differ only in their N- terminal, with the last 168 amino acids of the C-terminus encoded by exon 5, 6 and 7 being identical among the isoforms. The *RCAN1* gene has a tissue specific expression pattern with isoform 1 (RCAN1.1) being highly expressed in the central nervous system (CNS) and isoform 4 (RCAN1.4) being mostly expressed in heart muscle and fetal kidney [10], [11], [12], [13]. As an activating transcription factor 6 (ATF6) inducible gene, RCAN1 is activated under endoplasmic reticulum stress [14]. The RCAN1.1 isoform has been shown to be responsive to hyperglycemia, being implicated in hypoinsulinemia and diabetes, suggesting it plays a role in the deleterious effects observed after stress [15]. Our recent study has elucidated the molecular mechanism of RCAN1.1 transcription and has shown that RCAN1.1 expression can be regulated by the stress hormone glucocorticoid [16]. Furthermore we have shown that RCAN1.1 mediates oxidative stress-induced neurodegeneration in AD and DS pathogenesis, thus reinforcing the notion that RCAN 1.1 plays an important role in the neuronal response to stress [17]. An alternative promoter upstream of exon 4 of *RCAN1* gene responds to calcineurin-NFAT signaling pathway, controlling the expression of RCAN1.4 [18], [19]. Upregulation of RCAN1.4 was observed in peri-infarct cortex of stroke [20], yet ts role in neuronal function remains elusive [21], [22].

RCAN1 is phosphorylated at Ser^112^ by big mitogen-activated protein kinase 1 (BMK1) and dual specificity tyrosine-phosphorylation-regulated kinase 1A (DYRK1A), which is the priming site for the subsequent phosphorylation at Ser^108^ by glycogen synthase kinase 3(GSK3) [23], [24], [25], [26], [27]. Furthermore, phosphorylated RCAN1 is a substrate for calcineurin [26] and represses calcineurin-NFAT signaling pathway by inhibition of calcineurin both *in vitro* and *in vivo* [4], [28], [29], [30], thereby forming a negative feedback loop in RCAN1 isoform 4 regulation. RCAN1 has also been reported to regulate vesicle recycling via the inhibition of calcineurin [31], [32]. Recent studies have implicated RCAN1's involvement in neurodegeneration. RCAN1 interacts with Fragile X mental retardation protein and regulates both dendritic spine morphogenesis and local protein synthesis [33]. RCAN1.1 was seen to be deficient in Huntington disease and protective against mutant huntingtin toxicity [34]. Chronic expression of RCAN1.1 induces mitochondrial autophagy and dramatic degradation of mitochondria [35], [36]. Overexpression of RCAN1.1 *in vivo* leads to DS-like hippocampal deficits and impaired LTP, resulting in learning and memory deficits in transgenic mice [7], [37].A single extra transgenic copy of RCAN1 is sufficient to confer significant suppression of tumour growth in mice [38]. Our recent study showed that RCAN1.1 protein is markedly increased in DS and AD brains and its overexpression leads to neuronal apoptosis [16], [17], [35], [39]. However, the role of RCAN1.4 in neuronal apoptosis has not yet been explored.

Oxidative stress and disrupted calcium homeostasis are associated with neuronal death in AD [40], [41] and DS pathogenesis [42], [43], [44]. The effect of calcium overloading on RCAN1 regulation and its dysregulation on neuronal viability remains to be elucidated. In this study, we further characterized the RCAN1 exon 4 promoter and compared it with the exon 1 promoter. Our studies showed that the RCAN1 exon 1 promoter has higher activity in neuronal cell lines, whereas exon 4 promoter has a higher activity in non-neuronal cell lines. RCAN1.4 expression can be regulated by calcium overloading through NFAT signaling pathway. Exposure of cells to calcium stress exacerbates the neuronal death induced by RCAN1.4 overexpression. We further showed that caspase-3 is activated during neuronal death induced by RCAN1.4 overexpression and calcium overloading. This study further supports the involvement of RCAN1.4 in AD pathogenesis.

## Materials and Methods

### Cell Culture and drug treatment

C6 (ATCC CCL-107), N2A (ATCC CCL-131), SH-SY5Y(ATCC CRL-2266) and HEK293 (ATCC CRL-1573) cells were cultured in Dulbecco's modified Eagle's medium (DMEM) containing 10% FBS, 1 mM sodium pyruvate, 2 mM L-glutamine, 50 unit/ml penicillin G sodium and 50 µg/ml streptomycin sulfate (Invitrogen). All cells were maintained in a 37°C incubator containing 5% CO_2_. A23187 (Sigma) was dissolved in DMSO to 10 mM and diluted with complete cell culture medium to the final concentration of 2.5 uM.

### Plasmid construction-

pHA-NFAT, pDN-NFAT, pRIT-NFAT and pIL2-Luc plasmids were generous gifts from Drs. Anjana Rao and Alex Toker of Harvard Medical University [45], [46], [47], [48]. A fragment from –1650 bp to 46 bp (relative to transcription start site) from 5′UTR of RCAN1 exon 1 was amplified with primers 5′-gctagctagcaatatattgtgaacc and 5′-cacaagctttgtcagcagtctcccagc. The PCR fragments were cloned into pGL3-Basic to generate RCAN1 exon 1 promoter construct pRCAN1Luc-A. A fragment from −993 bp to +200 bp (relative to the transcription start site) from 5′UTR of RCAN1 exon 4 was amplified with 5′-ccgctcgagcatcgcagagcacttctc and 5′-cacaagcttgtgaaagcgctacagacc to generate RCAN1 exon 4 promoter construct pDE4Luc. Human RCAN1.4 coding sequence was amplified by RT-PCR with forward primer 5′-gcgcggatccgccaccatgcattttagaaactttaac and reverse primer 5′-gcgcgaattcgctgaggtggatcggcg. The amplified fragment was cloned into pcDNA4mycHisA to generate pcDNA4-RCAN1.4mychis.

### Transfection and luciferase assay

Cells were seeded one day before transfection and grown to approximately 70% confluence at the time of transfection. Cells were transfected with 0.5 µg of plasmid DNA in a well of 24 well plates with Lipofectamine 2000 (Invitrogen). The *Renilla* (sea pansy) luciferase vector pRluc was cotransfected to normalize the transfection efficiency. Cells were washed with PBS once 24–48 hours after transfection and lysed in 100 µl of 1× passive lysis buffer (Promega) for luciferase activity assay. 2 ul of lysates were mixed with the *firefly* luciferase assay reagent II and the luminescent signal was measured by the TD 20/20 luminometer (Turner Designs). Then 10 ul of Stop & Glo Reagent was added to the same tube and the luminescent signal from the *Renilla* luciferase was measured by the same luminometer. The *firefly* luciferase activity was normalized according to *Renilla* luciferase activity and expressed as relative luciferase units (RLU) to reflect the promoter activity [49].

### Antibodies and immunoblotting

The rabbit anti-RCAN1 polyclonal antibody DCT3 was raised against c-terminus of RCAN1 protein (RPKPKIIQTRRPEYTPIHLS) [16]. Cells were lysed in RIPA lysis buffer (1% Triton X100, 1% sodium deoxycholate, 4% SDS, 0.15 M NaCl, 0.05 M Tris-HCl, pH 7.2) supplemented with protease inhibitors (Complete, Boehringer Mannheim). The lysates were resolved by 12% SDS-PAGE for detecting RCAN1 and 16% tris-tricine PAGE for caspase-3. The immunoblotting was performed as described [50]. Rabbit anti-RCAN1 polyclonal antibody (1∶500 dilution) was used to detect RCAN1 expression. Pro-Caspase-3 and its cleaved form P20 were detected with a rabbit polyclonal antibody against 29-43 amino acid of caspase-3 (Sigma, Cat: C9598, 1∶1000). Mouse monoclonal antibody 9E10 was used to detect myc-tagged RCAN1.4. Internal control β-actin expression was analyzed using monoclonal anti-β-actin antibody AC-15 (Sigma, 1∶1000). LI- COR/Odyssey imaging system was used to quantify Western blots.

### RT-PCR

RNA was isolated from cells by TRI-Reagent (Sigma). PowerScript reverse transcriptase (Invitrogen) was used to synthesize the first strand cDNA from equal amount of the RNA sample following the manufacturer's instructions. The newly synthesized cDNA templates were amplified by Platinum *Tag* DNA polymerase (Invitrogen) in a 25 µl reaction. 20 to 35 cycles of PCR reaction were used to cover the linear range of the amplification. The isoform 4 was amplified by using 5′-gggtctgtagcgctttcac and 5′-cgcgtcgactggctgaggtggatcggcgtg. The primers were designed to specifically amplify RCAN1 isoform 4 without amplification of isoform 1. A pair of gene-specific primers 5′-tctggatcctcaccaccatggagaaggc and 5′-atactcgaggcagggatgatgttctg were used to amplify a 324 bp fragment of GAPDH as an internal control. The samples were further analyzed on 1.5% agarose gel. Kodak Image Station 1000 software (Perkin Elmer) was used to analyze the data.

### MTS assay

MTS assay was performed following the manufacturer's instructions (Promega). The MTS with PMS was added into culture media of cells and incubated for 4 hours in a 37°C incubator containing 5% CO_2_. Lysates were analyzed with a spectrophotometer at a wavelength of 490 nm (Multiskan Ascent, ThermoLab System).

### Apoptotic Cell Staining

TUNEL staining was performed following the manufacturer's instructions (Roche).Cells were rinsed twice with PBS and sealed in mount medium with DAPI (Vector lab). Apoptotic cells were analyzed under a fluorescence microscope (Axiovert 200. Germany).

### Statistical analysis

Data from three or more independent experiments were quantitatively analyzed by student's t-test or one-way ANOVA. Values were expressed as mean±S.E.M. P<0.05 was considered as statistically significant.

## Results

### Two alternative promoters distinctly control RCAN1 transcription

**Figure 1:**
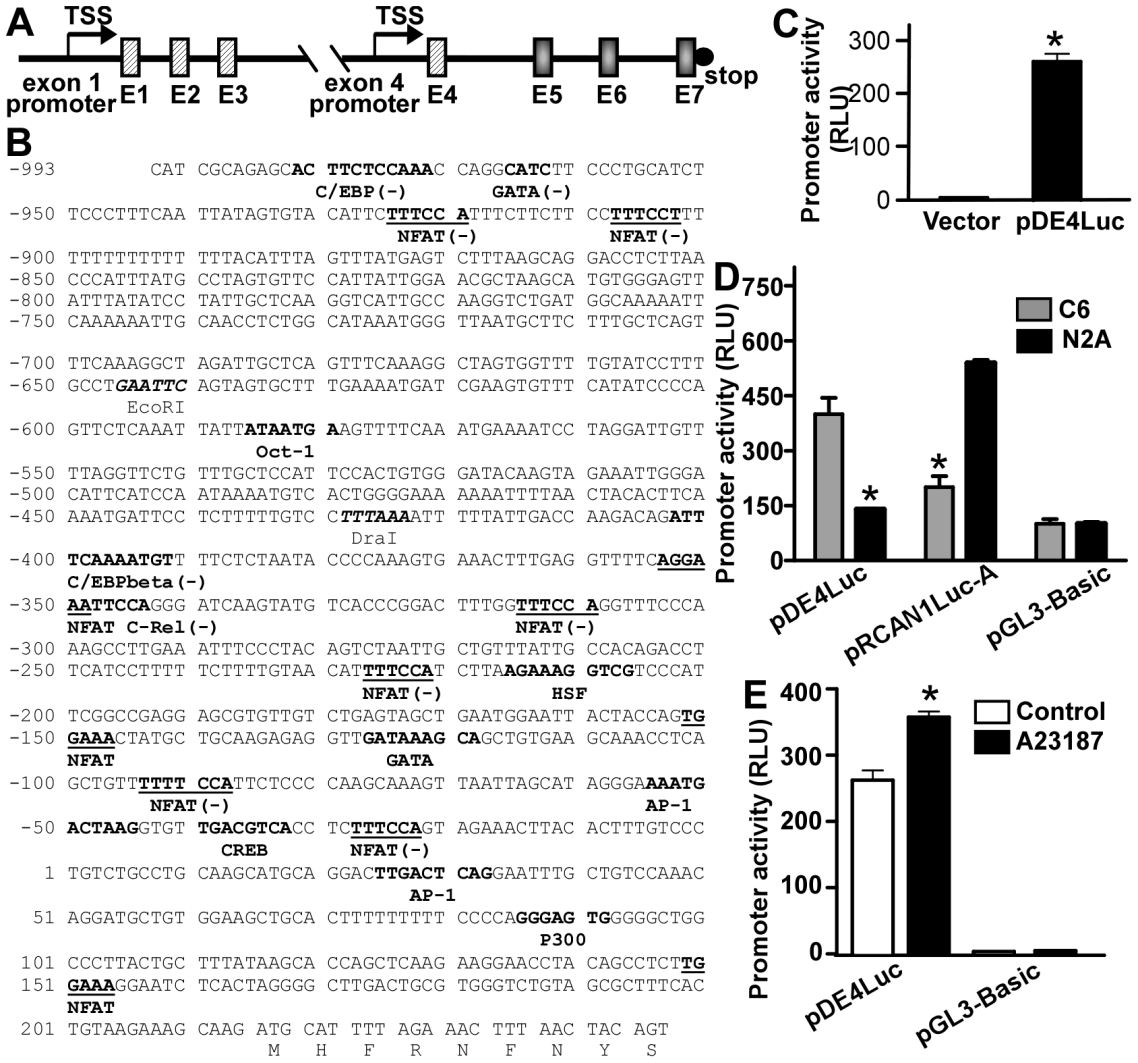
Two alternative promoters distinctly control RCAN1 expression. (**A**) Genomic organization of the *RCAN1* gene. *RCAN1* gene has seven exons and six introns. The first four exons are alternatively spliced and the last three exons are constitutive. There are two promoters and two translation initiation codons in the *RCAN1* gene, in the 5′UTR of exon 1 and 5′UTR of exon 4 respectively. TSS, transcription start site. E stands for exon. (**B**) Human *RCAN1* isoform 4 promoter sequence. A 1200 bp fragment of the 5′ flanking region of human RCAN1 exon 4 was amplified from a human genomic library. Thymine +1 represents the major transcription start site. Positions of some of the unique and common restriction enzymes are indicated in italics and boldface. Putative transcription factor binding sites are underlined in boldface. The codon of the first nine amino acids of exon 4 is indicated. (**C**) 1200-bp fragment upstream of the exon 4 had significant promoter activity. 1200-bp fragment upstream of the exon 4 was cloned into pGL3-Basic to generate the pDE4Luc luciferase reporter plasmid. pDE4Luc was transfected into HEK293 cells. pGL3-Basic was used as negative control. Luciferase activity was measured 24 hours after transfection. Values represent means ± SE (n = 4), *P<0.05 by student's t-test. (**D**) Compared to the RCAN1 exon 1 promoter pRCAN1Luc-A, RCAN1 exon 4 promoter pDE4Luc had a higher promoter activity in C6 cells but a lower activity in N2A cells. RCAN1 promoter constructs pDE4Luc and pRCAN1Luc-A were transfected into C6 and N2A cells. pGL3-Basic was used as negative control. Luciferase activity was measured 24 hours after transfection by a luminometer. *Renilla* luciferase activity was used to normalize transfection efficiency. Values represent means ± SE (n = 4), *P<0.05 by student's t-test. (**E**) Calcium ionophore A23187 significantly increased pDE4Luc activity. HEK293 cells transfected with pDE4Luc were treated with 2.5 µM A23187 for 12 hours. Luciferase assay was used to measure the promoter activity. pGL3-Basic was used as negative control. *Renilla* luciferase activity was used to normalize transfection efficiency. Values represent means ± SE (n = 4), *P<0. 05 by student's t-test.

The *RCAN1* gene contains seven exons and alternative splicing of the first four exons generates four different transcripts (Fig. 1A). Previously we characterized the RCAN1 promoter located upstream of the first exon and found that the transcription of RCAN1.1 isoform is tightly regulated [16], [17]. To examine whether RCAN1.4 expression is distinctly regulated by an alternative promoter at transcription level, a 1200-bp fragment from 5′UTR of *RCAN1* exon 4 was amplified by PCR and sequenced (Fig. 1B). To determine whether the 1200-bp fragment upstream of the exon 4 contains a promoter to activate transcription, this fragment was cloned into pGL3-Basic to generate the pDE4Luc luciferase reporter plasmid. Compared to the pGL3-Basic vector control, pDE4Luc transfected HEK293 cells display significant luciferase activity (262.52±29.44 RLU compared to 3.69±0.42 RLU, p<0.0001) (Fig. 1C), indicating that the 1200-bp fragment from the 5′ flanking region of *RCAN1* exon 4 contains a functional promoter.

Sequence analysis revealed several putative transcription factor binding sites in the RCAN1 exon 4 promoter, including NFAT, activator protein 1 (AP-1), GATA and CAAT enhancer binding protein (C/EBP) (Fig. 1B). Compared to the high GC content of RCAN1 exon 1 promoter region (74% GC in 600 bp upstream of translational start site) [16], the RCAN1 exon 4 promoter region has lower GC content (44% GC). Although the RCAN1 exon 4 promoter is a low-GC content promoter, there is no TATA-box in this promoter region, suggesting that RCAN1 exon 4 promoter, similar to the RCAN1 exon 1 promoter, is also a TATA-less promoter. The difference in GC content and transcription factor binding patterns suggests that the expression of RCAN1.1 and RCAN1.4 may be distinctly regulated.

It has been shown that RCAN1.1 is the predominant form expressed in the brain. To examine the expression of RCAN1.1 and RCAN1.4 in neuronal and glial cells and to elucidate the molecular mechanism underlying the cell specific expression of RCAN1.1 and RCAN1.4, we transfected the two RCAN1 promoter constructs pRCAN1Luc-A and pDE4Luc into rodent glial C6 and neuroblastoma N2A cells. Luciferase assay showed that the RCAN1 exon 4 promoter pDE4Luc had a much higher activity in C6 cell line than in N2A cell line, 399.3±45.01 vs. 139.7±1.70 RLU, respectively (p<0.005) (Fig. 1D). In contrast, the RCAN1 exon 1 promoter pRCAN1Luc-A had a markedly higher activity in neuronal cell line N2A than in glial cell line C6 cells, 538.7±8.60 vs. 200.4±30.01 RLU, respectively (p<0.001) (Fig. 1D). Furthermore, pDE4Luc has a higher promoter activity (399.3±45.01 RLU) than pRCAN1Luc-A in C6 cell line (200.4±30.01) (p<0.05) (Fig. 1D). In N2A cell line, pRCAN1Luc-A has a higher promoter activity (538.7±8.60 RLU) than pDE4Luc (139.7±1.70 RLU) (p<0.001) (Fig. 1D). These results suggest that different activities of two alternative RCAN1 promoters upstream of exon 1 and exon 4 account for the tissue specific expression of RCAN1.1 and RCAN1.4 in neuronal and glial cells.

Calcium overloading is a major pathophysiological alteration in stroke and RCAN1.4 is upregulated in peri-infarct region of stroke, indicating that calcium overloading may mediate RCAN1.4 upregulation [20]. To investigate how calcium overloading affects RCAN1 expression, HEK293 cells transfected with pDE4Luc were treated with the calcium ionophore A23187 at 2.5 µM. This divalent cation ionophore allows divalent cations to cross cell membranes and increases intracellular Ca^2+^ levels in intact cells. Luciferase assay showed that A23187 significantly increased pDE4Luc activity from 262.52±29.44 RLU to 357.38±17.6 RLU (p<0.005) (Fig. 1E). Taken together, these data demonstrated that two alternative promoters, localized upstream of RCAN1 exon 1 and exon 4, are responsible for the tissue-specific expression of RCAN1.1 and RCAN1.4. Calcium overloading can activate RCAN1 exon 4 promoter.

### Calcium overloading upregulates RCAN1 isoform 4 expression through activation of NFAT signaling pathway

**Figure 2:**
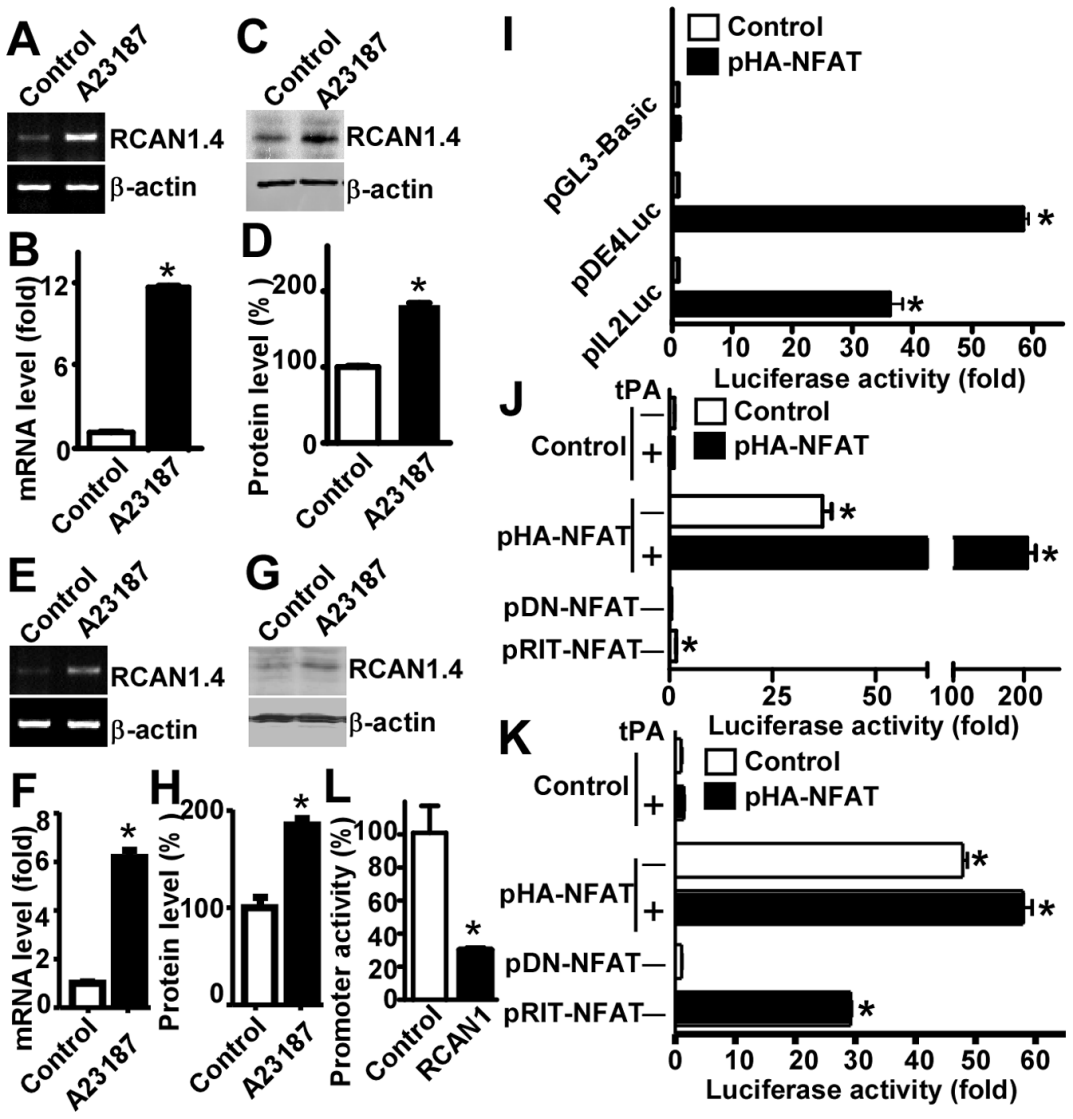
Calcium overloading upregulates RCAN1 isoform 4 expression through activation of NFAT signaling pathway. (**A**) Calcium ionophore A23187 increased *RCAN1* isoform 4 mRNA expression in HEK293 cells. A specific set of primers were used to amplify a *RCAN1* isoform 4 mRNA through RT-PCR. Samples were analyzed on 1.5% agarose gel. β-actin was used as internal control. (**B**) Quantification of (A). Values represent means ± SEM. n = 3, *P<0. 05 by student's t-test. (**C**) Calcium ionophore A23187 increased RCAN1 isoform 4 expression in HEK293 cells. RCAN1.4 protein expression is upregulated by A23187. HEK293 cells were treated with 2.5 µM A23187 for 12 hours. 150 ug cell lysates were separated in a 15% glycine SDS-PAGE gel. RCAN1 was detected with anti-RCAN1 antibody DCT3. β-actin detected with anti-β-actin antibody (Sigma, AC15) served as internal control. (**D**) Quantification of (C). Values represent means ± SE (n = 3), *P<0. 05 by student's t-test. (**E**) Calcium ionophore A23187 increased *RCAN1* isoform 4 mRNA expression in SH-SY5Y cells. SH-SY5Y cells were treated with 2.5 µM A23187 for 12 hours. A specific set of primers were used to amplify a *RCAN1* isoform 4 mRNA by RT-PCR. The samples were analyzed with 1.5% agarose gel. β-actin was used as an internal control. (**F**) Quantification of (E). Values represent means ± SE (n = 3), *P<0. 05 by student's t-test. (**G**) Calcium ionophore A23187 increased RCAN1.4 expression in SH-SY5Y cells. RCAN1.4 protein expression was upregulated by the treatment with A23187. SH-SY5Y cells were treated with 2.5 µM A23187 for 12 hours and 150 ug cell lysates were separated in a 15% glycine SDS-PAGE gel. RCAN1.4 was detected with anti-RCAN1 antibody DCT3. β-actin served as an internal control. (**H**) Quantification of (G). Values represent means ± SE (n = 3), *P<0. 05 by student's t-test. (**I**) NFAT significantly increased pDE4luc promoter activity. NFAT expression plasmid pHA-NFAT was co-transfected with RCAN1.4 promoter pDE4luc into HEK293 cells. pGL3-Basic was used as a negative control. And pIL2Luc that contains NFAT responsive elements was used as a positive control. Luciferase activity was measured 48 hours after transfection by a luminometer. Values represent means ± SE (n = 4), *P<0. 05 by student's t-test. (**J**) and (**K**) RCAN1 exon 4 promoter is activated by NFAT independent of AP1. HEK293 cells co-transfected with pHA-NFAT and pIL2Luc (**J**) or pDE4luc (**K**) were exposed to 100 nM tPA for 20 hours. NFAT dominant negative plasmid pDN-NFAT and mutant NFAT plasmid pRIT-NFAT that cannot interact with AP1 were also co-transfected. Luciferase activity was measured with a luminometer 48 hours after transfection. The X axis indicates fold increase of luciferase activity. Values represent means ± SE (n = 4), *P<0. 05 by student's t-test. (**L**) RCAN1 overexpression decreased RCAN1 exon 4 promoter activity. HEK293 cells were co-transfected with pRCAN1mychis and RCAN1 promoter constructs pDE4Luc. Luciferase activity was measured 48 hours after transfection. Values represent means ± SE (n = 4), *P<0. 05 by student's t-test.

We showed that treatment with the calcium ionophore A23187 activated RCAN1 exon 4 promoter activity (Fig. 1E). To investigate if calcium overloading could affect RCAN1.4 transcription and translation, RT-PCR and western blot assay were performed to examine the endogenous RCAN1.4 mRNA and protein levels in cells. A pair of RCAN1.4 specific primers were used to amplify *RCAN1* isoform 4 in cells exposed to calcium ionophore treatment. RT-PCR showed that *RCAN1.4* mRNA level was significantly elevated by more than 10 folds in HEK293 cells after 2.5 µM A23187 treatment for 12 hours (P<0.0001) (Fig. 2A and B). A23187 treatment significantly increased RCAN1.4 protein level (181.70±1.14% relative to the control, P<0.001) (Fig. 2C and D). To determine if calcium overload would have the same effects on neuronal cells, the same experiments were performed in human neuroblastoma SH-SY5Y cells. The levels of *RCAN1.4* mRNA and RCAN1.4 protein were significantly increased to 6.25±0.22 folds, P<0.01 (Fig. 2E and F) and 177.90±5.11%, P<0.01 (Fig. 2G and H), respectively after 2.5 µM A23187 treatment for 12 hours. These data demonstrated that RCAN1.4 expression is upregulated by calcium overloading.

Calcineurin-NFAT pathway is a major signaling pathway activated by calcium influx. Sequence analysis revealed there are nine putative NFAT sites with NFAT consensus sequence 5′-A/GGGAAA-3′ on the RCAN1 exon 4 promoter (Fig. 1A). To investigate whether upregulation of RCAN1.4 expression by calcium overloading is mediated by NFAT signaling pathway, NFAT expression plasmid pNFAT was co-transfected with RCAN1 promoter constructs into HEK293 cells. Plasmid pIL2Luc containing NFAT responsive elements served as a positive control [51]. Consistent with previous reports, NFAT overexpression in cells can markedly increase IL-2 promoter activity by approximately 36.22±4.38 folds compared to the control (p<0.001) and had no significant effect on luciferase activity of pGL3-Basic (P>0.05) (Fig. 2I). However, NFAT overexpression in HEK293 cells dramatically increased the luciferase activity of pDE4Luc by approximately 58.40±1.91 folds compared to the control (p<0.001) (Fig. 2I). These data suggest that RCAN1.4 expression is regulated by calcium-calcineurin-NFAT signaling pathway.

Cooperative binding of NFAT and AP-1 has been demonstrated on the promoter regions of many genes involved in immune response, such as cytokines and interleukins [51], [52]. To investigate if RCAN1 exon 4 promoter is also regulated by the cooperation of NFAT and AP-1, HEK293 cells co-transfected with pHA-NFAT and pDE4Luc or pIL2Luc were exposed to 100 nM tissue plasminogen activator (tPA), which can activate protein kinase C and strongly induce c-jun expression. Consistent with previous reports, tPA treatment strongly increased the IL-2 promoter activity in NFAT overexpressed cells from 29.69±3.59 folds to 204.6±24.45 folds (p<0.0001) (Fig. 2J). Furthermore, co-transfection of the dominant negative form of NFAT pDN-NFAT and the NFAT mutant pRIT-NFAT that can not interact with AP-1 decreased the IL-2 promoter activity to 0.34±0.015 and 1.29±0.72 folds respectively (P<0.0001) (Fig. 2J), suggesting that IL-2 expression is dependent on cooperation of NFAT and AP-1. However, in pDE4Luc transfected cells, RIT-NFAT mutant could still enhance the RCAN1 exon 4 promoter activity by 29.21±0.26 folds (p<0.001), though the increase was smaller than wild type NFAT (compared to 47.87±1.56 folds, p<0.001) (Fig. 2K). Furthermore, tPA treatment couldalso increase RCAN1 exon 4 promoter activity from 47.87±1.56 folds to 57.87±3.13 folds (p = 0.0012) (Fig. 2K). Co-transfection of dominant negative NFAT did not decrease the RCAN1 exon 4 promoter activity (1.07±0.06 folds compared to the control, p>0.05) (Fig. 2K). Taken together, these data suggest that the RCAN1 exon 4 promoter is independently activated by NFAT, which does not require the interaction with AP-1. This increase can be enhanced by AP-1 stimulator tPA, suggesting that there may be independent AP-1 binding sites on the RCAN1 exon 4 promoter.

Previous reports showed that RCAN1 can inhibit calcineurin activity, thereby it could form a negative feedback loop regulating RCAN1.4 transcription. To address this issue, RCAN1 expression construct pRCAN1mychis was co-transfected with RCAN1 promoters into HEK293 cells. Luciferase assay showed that RCAN1 overexpression in cells dramatically decreased RCAN1.4 promoter activity (30.56±1.24% relative to the control, p<0.01) (Fig. 2L). These data clearly demonstrate that RCAN1.4 expression is specifically regulated by calcium-calcineurin-NFAT signaling pathway in a negative feedback loop.

### RCAN1.4 overexpression exacerbates calcium overloading-induced-neuronal apoptosis

**Figure 3:**
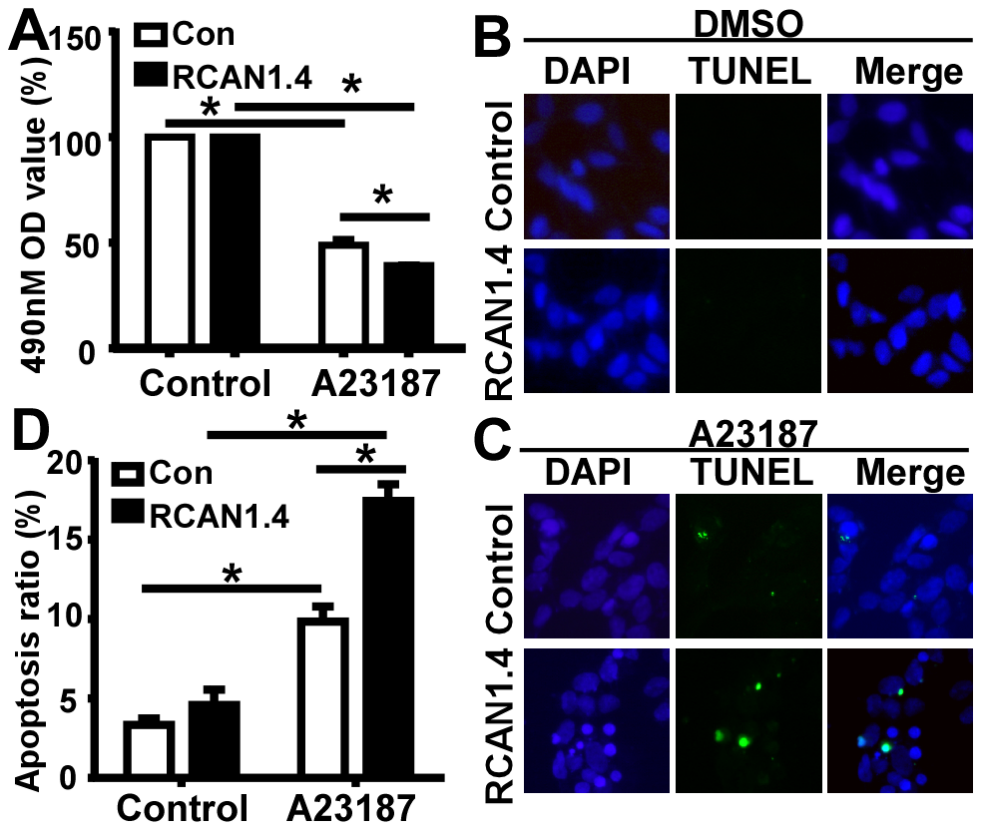
RCAN1.4 overexpression exacerbates calcium overloading-induced neuronal apoptosis. (**A**) RCAN1.4 overexpression reduced cell survival. SH-SY5Y cells transfected with empty vector pcDNA4mychisA or pcDNA4-RCAN1.4mychis were treated with 2.5 uM A23187. MTS assay was used to indicate the viability of SH-SY5Y cells. Values represent mean ± SEM, n = 3, *P<0. 05 by ANOVA. (**B**) and (**C**) RCAN1.4 overexpression exacerbated cell apoptosis. SH-SY5Y cells transfected with empty vector pcDNA4mychisA and pcDNA4-RCAN1.4mychis were treated with vehicle control solution (B) or 2.5 uM A23187and (C). TUNEL staining was used to indicate cell apoptosis (green color). Nuclei were counterstained with DAPI (blue color). (**D**) Quantification of (B) and (C). Values represent means ± SE (n = 3), *P<0. 05 by ANOVA.

Oxidative stress and calcium excess are associated with neuronal death in AD [40], [41]. Our previous study showed that RCAN1 isoform 1 is overexpressed in brains from DS and AD patients and RCAN1.1 overexpression in primary neurons potentiates oxidative stress-induced neuronal apoptosis [16], [17]. Although our data showed that calcium overload significantly increased the expression of RCAN1.4, the role of RCAN1.4 in neuronal apoptosis remains elusive. To investigate whether RCAN1.4 overexpression affects calcium overloading-induced neuronal apoptosis, human neuroblastoma SH-SY5Y cells with or without RCAN1.4 overexpression were exposed to 2.5 µM A23187 treatment for 12 hours. MTS assay was then carried out to evaluate neuronal viability. MTS assay showed that RCAN1.4 overexpression exacerbated calcium overloading-induced neuronal cell death, 39.16±0.15% vs. 48.86±1.54% in control cells (p<0.001) (Fig. 3A). TUNEL staining wasused to examine the neuronal apoptosis (Fig. 3B and C). Overexpression of RCAN1.4 in SH-SY5Y cells promoted calcium overloading-induced cell apoptosis, 17.44±0.61% vs. 9.84±0.55% in control cells (p<0.001 (Fig. 3D). Taken together, our data showed that RCAN1.4 overexpression can exacerbate the neuronal apoptosis induced by calcium overloading.

### Caspase-3 mediates neurotoxic effect of RCAN1.4 overexpression and calcium overloading

**Figure 4:**
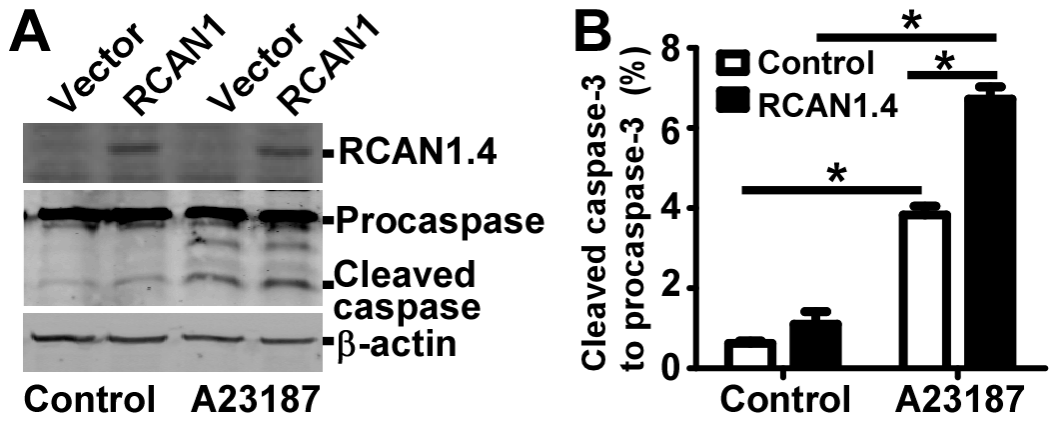
Caspase-3 mediates the neurotoxic effect of RCAN1.4 and calcium overloading. (**A**) RCAN1.4 overexpression increased caspase-3 activation. SH-SY5Y cells transfected with empty vector pcDNA4mychisA (Vector) and pcDNA4-RCAN1.4mychis (RCAN1) were treated with 2.5 uM A23187 for 12 hours. 100 µg cell lysates were separated in a 16% tricine SDS-PAGE gel. Procaspase-3 and cleaved caspase-3 were detected with anti-caspase-3 antibody from Sigma. Myc-tagged RCAN1.4 was detected by 9E10 antibody. β-actin served as loading control. (**B**) Quantification of (A). The ratio of cleaved caspase-3 to procaspase-3 was calculated. Values represent means ± SE (n = 3), *P<0. 05 by ANOVA.

Apoptosis has been implicated to play a major role in neuronal cell death in both AD and DS [44], [53], [54], [55]. Increased immunoreactivity of activated caspase-3 is observed in neurons of AD and DS patients [53]. Our recent study showed that RCAN1.1 overexpression in neurons activates caspase-3 [16], [17]. To investigate the molecular pathway of neuronal death induced by calcium overloading and the role of increased RCAN1.4 in this process, SH-SY5Y cells overexpressing RCAN1.4 and controls were exposed to 2.5 µM A23187 treatment for 12 hours. Cells were then lysed and cell lysates were separated in a 16% glycine SDS-PAGE gel. Immunoblotting of caspase-3 showed that RCAN1.4 overexpression increased caspase-3 activation by increasing the cleaved form of caspase-3 (Fig. 4A). Quantification of the Western blots showed that the ratio of cleaved caspase-3 to procaspase-3 was increased to 6.72±0.31% in RCAN1.4 overexpressing cells compared with 3.83%±0.22% in control cells with A23187 treatment (p<0.001(Fig. 4B), suggesting that RCAN1.4 overexpression potentiates calcium overloading-induced caspase-3 activation in human neuroblastoma cells.

## Discussion

Our recent studies showed that RCAN1.1 is overexpressed in cortical tissues from AD and DS patients, and it is upregulated by stress hormone glucocorticoid. Increased RCAN1.4 was reported in the peri-infarct area in cortical stroke [20]. To investigate the mechanism of RCAN1.1 and RCAN1.4 dysregulation, we further characterized human *RCAN1* gene promoters and found that *RCAN1* transcription is regulated by two alternative promoters. Differential activities of these two alternative promoters in different cells account for tissue-specific expression of RCAN1.1 and RCAN1. 4. The stress hormone glucocorticoid and calcium stress can upregulate RCAN1.1 and RCAN1.4 expression through alternative activation of these two promoters. Both HPA axis abnormality and calcium homeostasis disruption have been implicated in AD, which may be attributable to overexpression of RCAN1.1 and RCAN1.4. Nevertheless, overexpression of RCAN1 in DS may be attributed to on the trisomy of chromosome 21. Our study here further elucidated the molecular mechanism of RCAN1 transcription and advances our understanding of AD pathogenesis.

RCAN1 has been shown to be involved in many physiological and pathological processes, including cardiac valve development, cardiac hypertrophy, inflammation, angiogenesis, cancer and neurodegeneration. The vital role of RCAN1 in cell survival and death pathways indicates that RCAN1 expression level is tightly controlled. Our recent studies have shown that RCAN1.1 dysregulation and overexpression is implicated in neuronal apoptosis [16], [17]. In this study, we showed that RCAN1.4 overexpression promotes calcium overload-induced neuronal apoptosis. Furthermore, RCAN1.4 expression is regulated by a negative feedback loop, which could effectively prevent excessive production of RCAN1.4 and may help protect cells from unintentional cell death. Nevertheless, in pathological conditions such as DS and AD, RCAN1.4 expression may not be restrained by the negative feedback loop and could eventually lead to its overexpression and pro-apoptotic effect.

Memantine is a newly approved medication by European, US and Canadian regulatory agencies and also the only drug available for treatment of moderate to severe AD. Memantine is a non-competitive NMDA receptor antagonist that protects neurons from excessive calcium influx and cell death, suggesting calcium overloading is an important component in neuronal death and AD pathogenesis. In this study, we first demonstrated that calcium overloading upregulates RCAN1. 4 expression by activating the RCAN1.4 promoter through calcineurin-NFAT signaling pathway. Furthermore, we showed that overexpression of RCAN1.4 significantly exacerbates neuronal death induced by calcium overloading, which is mediated by the caspase-3 apoptotic pathway, suggesting that RCAN1.4 overexpression and calcium stress may work synergistically to facilitate neuronal death in AD. As RCAN1 knockout mice have no obvious phenotype [56], downregulation of RCAN1 may be devoid of severe side effects. Our study implies that downregulation of RCAN1 may protect neurons from apoptosis and therefore benefit AD patients.
